# TRANSITIONING TO WIDOWHOOD: THE ROLE OF SOCIAL INTEGRATION IN MODERATING WELL-BEING OVER TIME

**DOI:** 10.1093/geroni/igad104.1917

**Published:** 2023-12-21

**Authors:** Emily Kinkade, Heather Fuller

**Affiliations:** North Dakota State University, Fargo, North Dakota, United States; North Dakota State University, Fargo, North Dakota, United States

## Abstract

Prior studies indicate that widowhood may bring declines in both physical and mental health, but that social support may buffer against these declines. Social integration has also been found to alleviate widows’ depressive symptoms over time. However, less is known about whether social integration moderates the trajectory of well-being declines over longer periods of time post-widowhood. This study investigates whether social integration moderates the relationship between the transition to widowhood and changes in physical and mental well-being over time. A series of longitudinal multilevel models were run with data from participants (n=244-246) from five waves of the Social Integration and Aging study, collected every two years between 2013 and 2021. Depressive symptoms and subjective happiness were indicators of mental well-being, whereas basic and instrumental activities of daily living and total number of diseases were indicators of physical well-being. Social integration was assessed with the Social Integration in Later Life Scale (SILLS). Social integration moderated the relationship between widowhood and depressive symptoms such that increases in social integration resulted in greater reductions of depression scores over time among widows as compared to non-widows. Social integration similarly buffered against declines in physical functioning (i.e., increased basic activities of daily living) over time after widowhood occurred. No interaction effects of social integration on the relationship between widowhood and happiness or other physical well-being outcomes were found. These findings suggest social integration may be important for maintaining mental well-being and some aspects of physical health as older adults adjust to widowhood.

